# Clinical and Epidemiological Status of Leptospirosis in a Tropical Caribbean Area of Colombia

**DOI:** 10.1155/2018/6473851

**Published:** 2018-05-29

**Authors:** Vaneza Tique, Salim Mattar, Jorge Miranda, Misael Oviedo, Angel Noda, Eney Montes, Virginia Rodriguez

**Affiliations:** ^1^University of Cordoba, Tropical Biological Research, Monteria, Cordoba, Colombia; ^2^The Reference Laboratory of Spirochetes, Institute of Tropical Medicine “Pedro Kourí”, Cuba; ^3^Hospital San Jerónimo de Montería, Córdoba, Colombia

## Abstract

**Objective:**

To describe and analyze the clinical and epidemiological status in 28 confirmed cases of human leptospirosis at the main public hospital of Cordoba.

**Methods:**

Between 2012 and 2013, we conducted an active surveillance at the main hospital of Cordoba to establish the etiologic diagnosis of the undifferentiated tropical febrile illness (UTFI) cases. UTFI is defined as a fever without an infection focus in the initial physical examination or in basic laboratory tests. Patients in acute phase were accompanied by prodromal symptoms, including myalgia, arthralgia, headache, asthenia, chills, icterus, dyspnea, abdominal pain, rash, and nausea. Samples were collected on admission and at discharge. Clinical and epidemiological data were collected for each patient. Microscopic agglutination test (MAT) was performed.

**Results:**

The 28 leptospirosis cases presented the following gender distribution: male (n=24) and female (n=4). The duration of hospitalization was 10.39 days. The main symptoms and clinical manifestations were fever, headache and nausea, vomiting, and abdominal pain, all of which occurred in up to 60% of patients. Of the 28 cases studied, 4 were fatal. The most frequent infecting serogroups were Ballum and Canicola.

**Conclusion:**

Leptospirosis is a common cause of undifferentiated tropical febrile illness in Colombia; it is important to establish ongoing and accurate surveillance for acute febrile illness to facilitate the detection of cases of leptospirosis.

## 1. Introduction

Human leptospirosis is caused by bacteria that belong to the genus* Leptospira. *This genus comprises at least 22 species grouped into three categories containing pathogenic, intermediate, and saprophytic species. Currently, there are more than 250 named, potentially pathogenic serovars [1].

The disease results in high morbidity and considerable mortality in areas of high prevalence. It is estimated that around 10,000 cases of severe leptospirosis are hospitalized annually worldwide. The disease is usually endemic in areas with rainy season, humidity, close human contact with livestock, poor sanitation, and workplace exposure to the organism [2]. In recent years, a new trend in human leptospirosis outbreaks has been observed related to recreational activities among wildlife (a form of tourism that is becoming increasingly popular) and army expeditions, either for training or for combat-related purposes in similar environments [3]. A systematic literature review of Leptospirosis Burden Epidemiology Reference Group (LERG) reports an estimated global annual incidence of endemic and epidemic human leptospirosis ranging from 5 to 14 cases per 100,000. Endemic human leptospirosis rates have varied by region from 0.5/100,000 in Europe to 95/100,000 in Africa. Based on global data collected by International Leptospirosis Society surveys, the incidence was estimated to be 350,000–500,000 severe leptospirosis cases annually [4].

However, data emerging from prospective surveillance studies suggest that most human leptospiral infections in endemic areas may be mild or asymptomatic. Development of more severe outcomes likely depends on three factors: epidemiological conditions, host susceptibility, and pathogen virulence [5]. Fatality rates reported worldwide vary from 5% to 30%. This epidemiological picture is not reliable because in many areas the occurrence of the disease is not well documented. In addition, mild cases may not be diagnosed as leptospirosis [6]. Case fatality for pulmonary hemorrhagic syndrome and Weil's disease is more than 10% and 70%, respectively [5].

The Caribbean and Latin America, the Indian subcontinent, Southeast Asia, Oceania, and to a lesser extent Eastern Europe are the most significant foci of the disease, including areas that are popular travel destinations. According to Pappas et al., 2007, the annual incidence of leptospirosis cases per 100.000 in Latin America shows the following distribution: Costa Rica 67,2, Uruguay 25, Cuba 24,7, Brazil 12,8, Ecuador 11,6, Argentina 9, Venezuela 3,8, Chile 1,6, Colombia 1,6, and Panama 1,3. In Seychelles there were 432,1, Trinidad and Tobago 120,4, Barbados 100,3, Jamaica 78, Sri Lanka 54, Thailand 48,9, El Salvador 35,8, New Zealand 26, and Nicaragua 23,3 [3].

In Colombia, leptospirosis has been considered an event of mandatory notification to the National Surveillance System (SIVIGILA) since 2007 and has gained interest from health authorities, especially due to the increase in cases related to the rainy season and floods that have occurred in recent years. Knowledge about the characteristics of the disease is essential for improved surveillance and control of such events. Despite the increase in the notification of cases of leptospirosis per year in the country [7], no data are available regarding the current situation of the disease; most recent publications in Colombia have been focused on the characterization of outbreaks or have involved seroprevalence studies. In recent and previous study, we documented that leptospirosis was the most common cause of undifferentiated tropical fevers in this region [8].

The aim of the present study was to describe and analyze the clinical and epidemiological status in 28 confirmed cases of human leptospirosis at the main public hospital of Cordoba.

## 2. Material and Methods

### 2.1. Study Area

Cordoba is a region in the Caribbean Sea on the northern coast of Colombia. Monteria has an altitude range of 20-100 m above sea level, is covered by tropical dry forest vegetation, has an average temperature of 24°C, and receives between 1000 and 2000 mm^3^ of rain per year. Cordoba is a state devoted to agriculture and livestock production. Geographically the department of Córdoba can be divided into two regions based on ecosystems, separated by the mountains (Serranía) of San Jerónimo and influenced by its two main rivers: the Sinú and San Jorge. These two rivers, their valleys, and the mountain range that separates them create habitat conditions that could influence the ecology and distribution of* Leptospira* serogroups in the department of Cordoba.

### 2.2. Patients and Data Collection

Between 2012 and 2013, we managed an effective surveillance at the main hospital of Cordoba to determine the etiologic diagnosis of the undifferentiated tropical febrile illness (UTFI) cases. UTFI is defined as a fever without an infection focus in the initial physical examination or in basic laboratory tests [9]. In Colombia, UTFI are frequent infections; some of these strike during the year and in rainy or even during dry season (The Indian Society of Critical Care Medicine Tropical fever G). Throughout our analysis, there was no El Niño phenomenon nor floods and the occurrence of rain was normal. The examined area is not endemic for yellow fever or West Nile virus disease

Patients with acute phase were admitted to the emergency ward with febrile illnesses escorted by prodromal symptoms typical of UTFI infection, including myalgia, arthralgia, headache, asthenia, chills, icterus, dyspnea, abdominal pain, rash, and nausea. Patients were registered in a clinical trial for UTFI at the University of Cordoba. Serum samples were taken on admission and at discharge. Clinical and epidemiological data were collected for each patient during their hospital stay including age, sex, municipal origin, occupation, history of illness (date of onset of disease and date of admission), symptoms, physical findings, laboratory findings, and medical care. Case definition of leptospirosis was defined as specified by the National Health Institute of Colombia [7, 10].

### 2.3. Sampling

From each patient, we collected one acute phase and one convalescent phase (15-20 days after illness) peripheral venous blood sample. Seroconversion was described as negative serology which became positive in a convalescent serum sample; therefore the escalation of detectable antibodies (usually a fourfold titer increase) between the first and second sample was used as a definition in the present study.

### 2.4. Leptospirosis Diagnostics

Microscopic agglutination test (MAT) was performed according to the standardized protocol in the Reference Laboratory of Spirochetes, Institute of Tropical Medicine “Pedro Kourí”, Cuba. In the test were used 16 different serovars:* L. interrogans *Icterohaemorrhagiae Copenhageni strain M20,* L. interrogans *Icterohaemorrhagiae Icterohaemorrhagiae strain FGA;* L. interrogans *Canicola Canicola strain Hond Utrecht IV;* L. interrogans *Pomona Pomona strain Pomona;* L. borgpetersenii *Ballum Castellonis strain Castellon 3;* L. borgpetersenii *Sejroe Sejroe strain M 84;* L. interrogans *Sejroe Hardjo strain Hardjoprajitno;* L. interrogans *Sejroe Wolffi strain 3705;* L. interrogans* Pyrogenes Pyrogenes strain Salinem;* L. interrogans *Hebdomadis Hebdomadis strain Hebdomadis;* L. borgpetersenii* Tarassovi Tarassovi strain Perepelitsin;* L. interrogans* Australis Australis strain Ballico;* L. interrogans *Autumnalis Aautumnalis strain Akiyami A;* L. interrogans* Bataviae Bataviae strain Swart;* L. noguchii* Panama Panama strain CZ 214K;* L. kirschneri *Cynopteri Cynopteri strain 3522 C*; L. borgpetersenii J*avanica Javanica strain V Batavia 46;* L. kirschneri* Grippothyphosa Grippothyphosa strain Moskva V;* L. biflexa *Semaranga Patoc strain patoc I. Titers ≥1:160 were considered positive.

In addition, the Panbio®* Leptospira* IgM ELISA (Catalog E-LEP01M/E LEP01M05, Queensland, Australia) was used for serological screening. The test has demonstrated a diagnostic sensitivity of 96.5% and specificity of 98.5%.

### 2.5. Conventional PCR Assay

PCR was performed for 27 of the samples from blood anticoagulated with EDTA or sodium citrate. The PCR amplified a fragment of 146 bp of the lipoprotein gene* lipL32*, which is found only in pathogenic strains of* Leptospira* spp. The primers used were pfLp32-1 5′-TAGAATCAAGATCCCAAATCCTCC-3′ and pfLp32-2 5′-CCAACAGATGCAACGAAAGATCC-3′ for forward and reverse primers, respectively [11].

The PCR was performed in a 50 *μ*L volumes containing 0.125 U of* Taq *polymerase (QIAGEN, Germany), 1ul of each primer, PCR buffer 1X (dNTP 0.02 mM, MgCl_2_ 0.25 mM, KCl 0.025 M, Tris HCl 0.025 M, mg/mL), and 5 *μ*L of template DNA in a final volume of 50 *μ*L. PCR cycling conditions were carried out in automated MJ Research PTC-100TM thermal cyclers as follows: 1 cycle at 95°C for 3 min, followed by 40 cycles of 20 sec at 95°C, 30 sec at 60°C, and 30 sec at 70°C. PCR products were analyzed on a 2% agarose gel and visualized with SYBR safe in transilluminator (Biorad).

### 2.6. Differential Diagnosis

To establish other tropical prevalent pathologies in the studied area, serodiagnosis tests for malaria, hantavirus,* Rickettsia, Brucella*, Hepatitis A, and Hepatitis B were carried out [8].

### 2.7. Ethical Aspects

The investigation committee of the Institute of Tropical Biological Research of the University of Cordoba and Hospital San Geronimo of Monteria permitted the ethics protocol, and knowledgeable permission was achieved from all enrolled patients. Patients were anonymized using a numeric code. The study incorporated procedures, management and conservation of samples, and technical-administrative procedures for health research required by resolution 8430 of the Ministry of Health of Colombia, in 1993 [12], and declaration of Helsinki for ethical and medical research in human subjects [13].

## 3. Results

### 3.1. Sociodemographic and Geographic Characteristics

**Figure 1:**
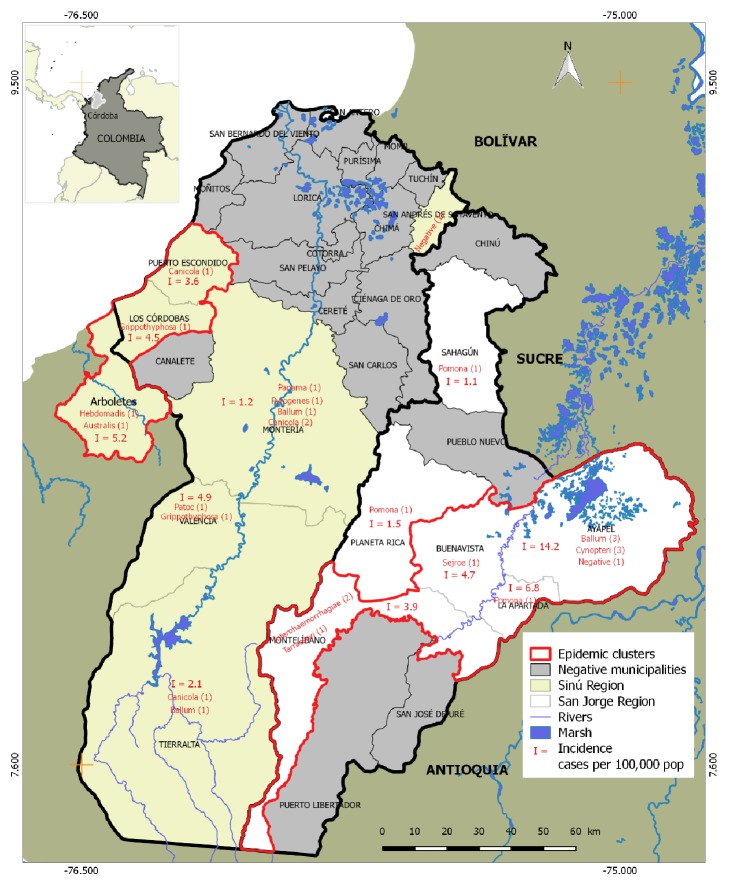
Distribution of leptospiral serogroups in the department of Córdoba.

The 28 leptospirosis cases presented the following gender distribution: male (n=24) and female (n=4). The mean age was 26.7 years. Six people were linked to farming, while the other 22 were engaged in other activities. Of the 28 cases, 14 belonged to each of the two geographic regions, with 11 men and 3 women; 4 people died, two in each region. The age of those affected was statistically the same in the two regions, 27.7 years in San Jorge region and 25.8 years in Sinú river region.  Figure 1 shows the serogroups in the two studied regions. The prevalence of serogroups was different; in the San Jorge region were identified Pomona (n=3), Ballum (n=3), Cynopteri (n=3), Icterohaemorrhagiae (n=2), Sejroe (n=1), Tarrassovi (n=1), and 1 negative. Conversely, in the Sinú region the distribution was Canicola (n=4), Grippothyphosa (n=2), Australis (n=1), Patoc (n=1), Hebdomadis (n=1), Pyrogenes (n=1), Panama (n=1), Ballum (n=2), and 1 negative. Ballum was the only serogroup present in the two regions, in San Jorge region (n=3) and in Sinú region (n=2), and the Ballum was most prevalent in the study.

Figure 1 shows the distribution of cases of leptospirosis serogroups and annual incidence in the different affected municipalities. The analysis of the distribution of annual incidence (cases per 100,000 inhabitants) identified two clusters with epidemic activity, one in the San Jorge region and another in the Sinú region. The San Jorge conglomerate was formed by the municipalities of Ayapel (I=14.2), La Apartada (I=6.8), Buenavista (I=4.7), and Montelibano (I=3.9). The Sinú conglomerate was made up of the municipalities of Arboletes (I=5.2), Los Córdobas (I=4.5), Puerto Escondido (I=3.6), and most likely Valencia (I=4.9). In the conglomerate of the regions of San Jorge and sinu the serogroups are present.

### 3.2. Clinical Presentation of the Disease

The average duration of illness was 6.7 days and the duration of hospitalization was 10.4 days. The main symptoms and clinical manifestations are shown in Table 1. The main clinical symptoms were fever, headache, and nausea. Vomiting and abdominal pain presented in up to 60% of patients. Other important symptoms were myalgia, arthralgia, chills, jaundice, hepatomegaly, coluria, mucocutaneous pallor, and dyspnea. The main clinical manifestations were thrombocytopenia, lymphocytosis, neutrophilia, and leukocytosis. The clinical picture was statistically the same in patients from the two geographical regions; minor clinical manifestations (not shown) were also statistically the same between the two geographical regions. Hemorrhagic manifestations were infrequent; only 5 patients presented with gingivorrhagia. Two patients presented with malaria coinfection and another with dengue and hantavirus.

The major antibiotics administered to the patients were as follows: 13 (46.4%) were given ceftriaxone, 4 (14.3%) clindamycin, 4 (14.3%) vancomycin, and 3 (10.7%) penicillin. Acetaminophen was administered to 18 (64.3%) of the patients.

Three categories were established taking into account the compatibility of the leptospiral diagnostic leading to the following final diagnoses: (i) high compatibility with leptospirosis or hemorrhagic fevers in 12 cases; (ii) incompatibility in 6 cases; (iii) slight compatibility in 10 cases. Of 28 cases studied only 4 were classified with a diagnosis of leptospirosis at discharge; one of the patients who presented with IgM ELISA positive and MAT negative died.

### 3.3. Lethal Cases of Leptospirosis in Córdoba

Of the 28 cases studied, 4 were fatal (Table 2). One of these cases was negative by MAT and positive by IgM but he presented signs and symptoms consistent with the disease. Four cases were considered febrile syndrome or icteric hemorrhagic syndrome and only one of them was diagnosed as leptospirosis.

### 3.4. Conventional PCR Results

We attempted to amplify a fragment of the lipL32 gene; however, all blood samples were negative.

## 4. Discussion

The present study shows a cohort of 100 patients who were enrolled in a tropical febrile disease trial; 28 patients were diagnosed with leptospirosis by laboratory tests of MAT and ELISA IgM. Leptospirosis is a major concern because of its high prevalence of 28% in this region of the country [8].

In Colombia since 2009, 16.989 cases have been notified to SIVIGILA but only 7.481 (44%) have been confirmed. A national incidence of 1.15/100.000 has been established. In Córdoba state in 2016 were reported 55 cases; only one of them was confirmed as fatal [7]. These data demonstrate the importance of studying this disease, particularly in endemic and tropical areas. (Table 3).

The highest incidence of leptospirosis was in the area of Ayapel (San Jorge region), where there is a large lagoon with an important agricultural activity and artisanal fishing. It is likely that being a flood prone area resulted in increased incidence of leptospirosis. In addition, it is an area lacking basic public services, which contributes to the proliferation of tropical diseases. The area is also affected by contamination with heavy metals from illegal mining and spraying of pesticides. The next highest frequency of leptospirosis was Monteria, an important urban area with semirural areas and nearby rivers. The socioeconomic conditions of patients affected in Monteria also make them prone to leptospirosis, since there are no public services such as sanitary sewage and potable water.

Some studies have reported the circulation of* Leptospira* spp. in Colombia, from the late 1960s, with seroprevalence ranges from 3.9% to 35.8%. However, an accurate situation of the disease is unknown in most regions of Colombia [14–16]. In a study carried out in Villavicencio state of Meta (Colombia) in the south east and near the Venezuelan border, the seroprevalence of* Leptospira *spp. in an apparently healthy population and in groups at risk was determined. In the low risk group the seroprevalence was 5.2% and 19% for groups at risk. Three factors were found to be associated with higher seroprevalence: rural social level, having a pet dog, and contact with rodents in the workplace [17]. Although our seroprevalence is higher (28%), the same factors associated with the increase of seroprevalence were found in the present study in patients from rural areas.

In a study carried out during 2007 and 2008, sera were collected from 220 nonmalarial acute febrile and convalescent patients from the rural and urban zones of Necocli, Turbo, and Apartado, areas close to our study area [18]. These authors found a frequency of infection for leptospirosis of 14.1%, as well as 12 coinfection cases of leptospirosis-dengue and one of leptospirosis-rickettsiosis-dengue. Although the frequency of infection for leptospirosis in our study was double (28%), we found two coinfections, namely, leptospirosis-malaria and leptospirosis-dengue-hantavirus. These coinfections could be common in this region of the country due to the endemicity of tropical diseases as dengue, rickettsiosis, and hantavirus.

Seroprevalence studies may represent a good indicator of the circulation of the pathogen. In that sense, in Uraba, Antioquia near the Panama border, a seroprevalence of 12.5% was detected [14]. No differences were observed according to race, gender, occupation, age, living conditions, or time living in the area. Our results are higher than others in a similar area near the present study. Other studies in the same region have found that the practice of barefoot walking in domestic environments represented a 4.27-fold higher risk for leptospirosis [19]. Although the present study did not evaluate these aspects, barefoot walking is a common practice in the study area; it is necessary to carry out follow-up studies in these municipalities to evaluate this factor.

In a study carried out in Tunja (Colombia), a prevalence of 21.7% in humans was found [20]. In the same study, in the canine population a seroprevalence of 67.2% was found; in another study, in three municipalities of Tolima (Colombia) a seroprevalence of 21.4% was found [21]. These findings suggested that dogs are potential reservoirs of* Leptospira *in these areas of the country.

MAT is usually positive 10 to 12 days after the onset of the first symptoms and clinical signs, but seroconversion may occur as early as 5-7 days after the onset of the disease. The antibody response can be delayed if antibiotic therapy is started before the test is performed. The antibody titer should be interpreted in light of the date of collection of the sample in relation to the first clinical signs; the evolution of antibody titers between the two or three successive samples; the causal serogroup; the treatment given [6]. In the present study all patients were positive for ELISA (IgM) and two were negative for MAT. 39% (11/28) of the patients presented a seroconversion with an increase in titers up to four times. Caution is necessary in the interpretation of serological data. Several factors must be taken into consideration, including the technique used, the serogroup involved, the chronological order of the samples taken during the disease, and the treatment with antibiotics if any. The specific gender test tends to be positive earlier in the course of the disease than the MAT.

The presence of cases of Leptospira of the present study is corroborated with the study in Córdoba, where a high seroprevalence of 75.8% in humans is established [22]. They also detected seven L. interrogans sensu lato strains isolated from different sources (pigs, dogs, and water). High seroprevalence in humans, concomitant to isolation of strains, demonstrates that, in Cordoba, transmission exists among animals, the environment, and humans [22].

Regarding clinical symptoms, signs, hematological alterations, and hemorrhagic presentations, there were no remarkable findings to allow us to differentiate among the frequent tropical acute infectious diseases found in that area. It is difficult to differentiate between leptospirosis, dengue, and malaria due to the overlap in clinical symptoms, signs, hemorrhagic presentations, and hematological alterations (Table 1). For example, thrombocytopenia was found in 80% of patients with dengue and 60% of those with leptospirosis. Epistaxis, petechiae, gingival hemorrhage, leukopenia, neutrophilia, and anemia were seen without a remarkable predominance in dengue and leptospirosis. We studied only 28 patients and it may be possible that some pathologies were unrepresented, making it difficult to establish a definitive conclusion for this disease.

Leptospirosis is among the leading zoonotic causes of morbidity worldwide and accounts for numbers of deaths, which approach or exceed those for other causes of hemorrhagic fever. Highest morbidity and mortality occur in resource-poor countries, which include regions where the burden of leptospirosis has been underappreciated [5]. The lethality in our study was 14%, but of the 4 patients who died, only 1 was diagnosed with leptospirosis, demonstrating the complexity of diagnosis due to the similarity of the clinical presentation of the cases that can be confused with dengue, malaria, hantavirus, arenaviruses, rickettsiosis, and salmonellosis. Regarding the antibiotics administered to the patients, only 2 (57%) received ceftriaxone and penicillin, both with activity against* Leptospira*, and the remaining 2 were treated erroneously.

Despite some limitations such as the small number of patients that do not allow definitive conclusions, the study allowed us to define some clinical and epidemiological features of patients with leptospirosis in the state of Córdoba. It also highlighted some failures in the clinical diagnosis and management of cases of leptospirosis.

In conclusion, it is important to establish ongoing and accurate surveillance for acute febrile illness to facilitate the detection of cases of leptospirosis. Early recognition and treatment of patients have been shown to reduce the duration and severity of illness. Surveillance is also useful for identifying outbreaks early where mass prophylaxis could be considered, especially in areas with high numbers of cases and limited access to healthcare [23]. These results will guide interventions in health and environmental control in the area of this disease forgotten or confused with other endemic febrile syndromes such as dengue or malaria.

## Figures and Tables

**Table 1:** Main symptoms, clinical manifestations, and laboratory findings of patients with leptospirosis.

Main symptoms and clinical manifestations	Frequency	%	Frequency by area		p-value
			San Jorge	Sinú	
Fever	28	100,0	14	14	1.000
Headache	20	71.4	11	9	0.403
Nausea	19	67.9	9	10	0.686
Vomiting	18	64.3	9	9	1.000
Abdominal pain	17	60.7	11	6	0.053
Myalgia	16	57.1	9	7	0.445
Arthralgia	16	57.1	9	7	0.445
Chills	15	53.6	9	6	0.256
Jaundice	13	46.4	6	7	0.705
Hepatomegaly	13	46.4	5	8	0.256
Coluria	11	39.3	5	6	0.699
Mucocutaneous pallor	10	35.7	5	5	1.000
Dyspnea	9	32.1	5	4	0.686

Thrombocytopenia	20	71.4	12	8	0.094
Lymphocytosis	11	39.3	6	5	0.699
Neutrophilia	11	39.3	6	5	0.699
Leukocytosis	8	28.6	4	4	1.000

**Table 2:** Lethal cases of leptospirosis in Córdoba.

Id/Gender Years Procedence	Devol	Dhos	Clinical manifestations	Blood count	IgM (Value )	MAT 1 (title)	MAT 2 (title)	MAT Results (serogroups)	Antibiotic therapy	Diagnosis at admission	Discharge diagnosis
1/F 14 Puerto Escondido.	7	13	Fever, headache, nausea, jaundice, vomiting, hyporexia, asthenia, coluria, cough abdominal pain, diarrhea, abdominal examination, mucocutaneous pallor, dyspnea, pleural effusion, ascites, hepatomegaly, tachypnea.	Leukocytosis, mild anemia, grade I thrombocytopenia	30.1 Positive	160	640	canicola	DA, CN, AMP, SAM	Viral hepatitis, abdominal pain	Hepatic failure, hepatitis under study

2/M 14 San Andrés Sotavento.	4	2	Fever, headache, nausea, vomiting, chills, abdominal pain, diarrhea, abdominal examination, convulsions, mucocutaneous pallor, dyspnoea, pleural effusion, ascites, hepatomegaly, splenomegaly, tachycardia, tachypnea, algidity, hypotension, bilateral infiltration, hypoventilation.	Neutrophilia, mild anemia, severe leukopenia, grade III thrombocytopenia.	31.6 Positive	0	0	Negative	DA,CRO,ACET	Dengue	Leptospirosis

3^*∗*^/M 47 Montelibano	10	6	Fever, headache, nausea, vomiting, chills, retroocular pain, abdominal pain, diarrhea, abdominal examination, seizures dyspnea, pleural effusion, tachypnea. Crepitos.	Lymphocytosis, mild anemia, mild leukopenia, grade II thrombocytopenia.	14.7 Positive	320	640	icterohaemorrhagiae	DA, Antimalarico.	Malaria	Malaria

4/M 45 Ayapel	5	38	Fever, headache, nausea, vomiting, chills, asthenia coluria, hyperconjunctival injection, abdominal pain, abdominal examination, mucocutaneous pallor, dyspnea, pleural effusion, asitis, hepatomegaly, tachycardia, tachypnea, hypoventilation.	Leukocytosis, neutrophilia, grade II thrombocytopenia.	27.8 Positive	40	160	Ballum	CN,SAM,MEM.	Hemorrhagic icteric syndrome	Unspecified jaundice, unspecified renal failure

F: female, M: male, Devol: days of evolution at admission, Dhosp: days of hospitalization, ICU: intensive care unit, DA: clindamycin, CN: gentamicin, AMP: ampicillin, SAM: ampicillin & sulbactam; CRO: ceftriaxone, MEM: meropenem, ACET: acetaminophen. ^*∗*^Patient with underlying arterial hypertension, required ICU admission, coinfection with malaria.

**Table 3:** Total cases of leptospirosis reported by national public health surveillance system, Colombia, 2009-2017.

Years	Number of reported cases	Suspected	Confirmed	(%)
2009	1815	827	988	54.44
2010	2261	1026	1235	54.62
2011	2478	1237	1191	48.06
2012	1986	943	1043	52.52
2013	1940	1073	867	44.69
2014	2305	1368	846	36.70
2015	2007	1225	782	38.96
2016	2197	1635	529	24.07
2017^*∗*^	113	-	-	-
Total^*∗∗*^	16989	9334	7481	44.03

Source: SIVIGILA, National Institute of Health, Colombia, 2009-2017. *∗* Leptospira cases to epidemiological week 12 of 2017. *∗∗*Not including 2017.

## Data Availability

The data used to support the findings of this study are available from the corresponding author upon request.
